# CD8 and CD4 Epitope Predictions in RV144: No Strong Evidence of a T-Cell Driven Sieve Effect in HIV-1 Breakthrough Sequences from Trial Participants

**DOI:** 10.1371/journal.pone.0111334

**Published:** 2014-10-28

**Authors:** Kalpana Dommaraju, Gustavo Kijak, Jonathan M. Carlson, Brendan B. Larsen, Sodsai Tovanabutra, Dan E. Geraghty, Wenjie Deng, Brandon S. Maust, Paul T. Edlefsen, Eric Sanders-Buell, Silvia Ratto-Kim, Mark S. deSouza, Supachai Rerks-Ngarm, Sorachai Nitayaphan, Punnee Pitisuttihum, Jaranit Kaewkungwal, Robert J. O'Connell, Merlin L. Robb, Nelson L. Michael, James I. Mullins, Jerome H. Kim, Morgane Rolland

**Affiliations:** 1 US Military HIV Research Program, Walter Reed Army Institute of Research, Silver Spring, Maryland, United States of America; 2 Henry Jackson Foundation, Bethesda, Maryland, United States of America; 3 Microsoft Research, Los Angeles, California, United States of America; 4 Department of Microbiology, University of Washington, Seattle, Washington, United States of America; 5 Vaccine and Infectious Disease Division, Fred Hutchinson Cancer Research Center, Seattle, Washington, United States of America; 6 Armed Forces Research Institute of Medical Sciences, Bangkok, Thailand; 7 Thai Ministry of Public Health, Nonthaburi, Thailand; 8 Royal Thai Army Component, Armed Forces Research Institute of Medical Sciences, Bangkok, Thailand; 9 Faculty of Tropical Medicine, Mahidol University, Bangkok, Thailand; University of Alabama, United States of America

## Abstract

The modest protection afforded by the RV144 vaccine offers an opportunity to evaluate its mechanisms of protection. Differences between HIV-1 breakthrough viruses from vaccine and placebo recipients can be attributed to the RV144 vaccine as this was a randomized and double-blinded trial. CD8 and CD4 T cell epitope repertoires were predicted in HIV-1 proteomes from 110 RV144 participants. Predicted Gag epitope repertoires were smaller in vaccine than in placebo recipients (p = 0.019). After comparing participant-derived epitopes to corresponding epitopes in the RV144 vaccine, the proportion of epitopes that could be matched differed depending on the protein conservation (only 36% of epitopes in Env vs 84–91% in Gag/Pol/Nef for CD8 predicted epitopes) or on vaccine insert subtype (55% against CRF01_AE vs 7% against subtype B). To compare predicted epitopes to the vaccine, we analyzed predicted binding affinity and evolutionary distance measurements. Comparisons between the vaccine and placebo arm did not reveal robust evidence for a T cell driven sieve effect, although some differences were noted in Env-V2 (0.022≤p-value≤0.231). The paucity of CD8 T cell responses identified following RV144 vaccination, with no evidence for V2 specificity, considered together both with the association of decreased infection risk in RV 144 participants with V-specific antibody responses and a V2 sieve effect, lead us to hypothesize that this sieve effect was not T cell specific. Overall, our results did not reveal a strong differential impact of vaccine-induced T cell responses among breakthrough infections in RV144 participants.

## Introduction

The RV144 trial showed modest efficacy in preventing HIV-1 infection with an estimated 31% reduction in HIV-1 infections in the vaccine arm (modified intent to treat population, p = 0.04) [1]. Among the 16,402 adult Thai participants, 125 HIV-1 infections were diagnosed following enrollment. We sequenced HIV-1 near full-length genomes from plasma samples collected at the time of HIV-1 diagnosis in 121 subjects [2]. Phylogenetic analyses showed that 110 of these infections were caused by CRF01_AE viruses.

A genetic analysis spurred by the identification of a correlate of risk of infection linked to Env-V2, focused on this Env segment and identified two signatures that distinguished viruses from vaccine and placebo recipients [2]. Viruses derived from vaccine recipients could be differentiated from those from placebo recipients at Env positions 169 and 181, which are contact sites for V2-specific antibodies including some derived from RV144 participants [3]. Genetic signatures in V2, together with the identification of binding antibodies directed against V2 as a correlate of risk of HIV-1 infection [4], suggest that anti-V2 antibodies may have played a role in the protection conferred by the RV144 vaccine.

It is plausible that Env-V2 was not the sole viral determinant impacted by vaccine-induced immune responses. One possible path to explore is the potential role of T-cell mediated immune responses. Analyses performed on samples collected 6 months after the final immunization showed Gag or Env IFN-γ ELISpot responses in 20% of vaccinees vs 7% of placebo recipients [1]. Intracellular cytokine staining assays showed no difference between vaccine and placebo recipients for CD8 responses (Gag CD8: 7% of responders; Env CD8: 11% and 14% of responders among vaccine and placebo recipients, respectively) or for Gag CD4 responses (1% vs 0% of responders), while Env CD4 responses were significantly more frequent in vaccine than in placebo recipients (34% vs 4%, respectively) [1].

To obtain insights on the impact of T cell immunity on founder HIV-1 sequences, potential CD8 and CD4 epitopes can be predicted *in silico* based on sequence motifs matched by class I and II HLA alleles [5], [6]. In this study, we performed CD8 and CD4 epitope predictions based on each subject’s HLA genotype and HIV-1 proteome sequence(s) using the same methods as in the analysis of breakthrough infections in the Step/HVTN502 trial [5],[6], which we have expanded to include comparisons of epitope predictions based on evolutionary distances and predicted affinity binding. We analyzed subject-derived epitope predictions as a function of the epitopes predicted in the RV144 vaccine inserts to investigate whether we could identify features distinguishing the vaccine and placebo group.

## Materials and Methods

### Ethics Statement

The RV144 clinical vaccine trial was registered with ClinicalTrials.gov and assigned the registration number NCT00223080 (Supporting File S1). The protocol was approved by the ethics committees of the Ministry of Public Health, the Royal Thai Army, Mahidol University, and the Human Subjects Research Review Board of the U.S. Army Medical Research and Materiel Command. It was also independently approved by the World Health Organization and the Joint United Nations Program on HIV/AIDS and by the AIDS Vaccine Research Working Group of the National Institute of Allergy and Infectious Diseases at the National Institutes of Health.

Written informed consent was obtained from all volunteers, who were required to pass a written test of understanding. The consent procedure was approved by the Ethics committees and IRBs listed above.

The RV144 trial was double-blinded and randomized, enrolled 16,402 participants and took place in Thailand between October 2003 and September 2009; the results of the trial were reported by Rerks-Ngarm and colleagues [1], and further details on immune correlates of risk of infection were reported by Haynes and colleagues [4].

### HIV-1 sequence data

For RV144 participants who became HIV-1-infected during the RV144 trial (diagnosed between 14 June 2004 and 12 February 2009), HIV-1 near full-length genomes were sequenced from single RNA templates corresponding to plasma samples collected at the time of HIV-1 diagnosis (GenBank accession numbers JX446645–JX448316). Sequences from the 110 subjects who were infected with CRF01_AE viruses were translated to amino acid (AA) sequences, using only sequences with open reading frames.

Vaccine insert sequences corresponded to two lab strains of HIV-1 subtype B and two CRF01_AE viruses isolated in Thailand in 1990 and 1992. The ALVAC-HIV canarypox prime [vCP1521] is a chimeric construct that concatenates *gag* and *pro* of HIV-1 subtype B (strain LAI) with gp120 of CRF01_AE (strain 92TH023) fused to a 28-AA-long segment of the transmembrane-anchoring portion of gp41 HIV-1-B strain LAI (HXB2 position AA 684∶711 of HXB2 gp160). The AIDSVAX B/E boost was composed of two gp120 proteins with N-terminal truncations (HIV-1 protein started at AA42 of HXB2 gp160): one protein was HIV-1 subtype B (strain MN) and one was HIV-1 CRF01_AE (strain CM244).

### HLA genotyping

High-resolution typing of class I and II HLA was performed by DNA sequence-based typing (SBT) and by the sequence-specific oligonucleotide probe (SSOP) method, with concordant results. Class I SBT was carried out by PCR amplification and subsequent dye terminator nucleotide sequencing of exons 2 and 3, with ambiguous types being resolved to four digits using the dbMHC SBT interpretation interface [7] (http://www.ncbi.nlm.nih.gov/projects/gv/mhc/). Class II SBT were genotyped in the CLIA/ASHI accredited lab of William Hildebrand at the University of Oklahoma Health Sciences Center using in-house PCR and sequencing methodologies. The entirety of exon 2 was DNA sequenced for all class II loci with additional exons DNA sequenced for DQB1 (exon 3), DQA1 (exon 3), and DPB1 (exons 3 & 4). DNA sequence analysis and HLA allele assignment were performed with Assign-SBT v3.5.1 software (Conexio Genomics). The HLA database for allele assignment was updated with IMGT release 3.0.0 May 5th 2010. Any ambiguous types that remained following DNA Sequence Based Typing were resolved to 4-digits using the PEL-FREEZ UNITRAY SSP, Life Technologies. SSOP was conducted using the LABType SSO Class I HD system (One Lambda, Canoga Park, CA), which is based on Luminex xMAP technology, and results were interpreted using the accompanying HLA Fusion 2.0.0 software. HLA types are reported according to the IMGT/HLA nomenclature (version 3.7.0, http://www.ebi.ac.uk/imgt/hla/ambig.html).

### CD4^+^ and CD8^+^ T cell epitope predictions

CTL epitope predictions were done with the NetMHCpan 2.4 Server, which predicts binding of peptides to each subject’s HLA genotype using artificial neural networks [8] (http://www.cbs.dtu.dk/services/NetMHCpan/). Predictions were done for 9-mers, because it is the favored length for binding (predictions for other lengths are made from approximations based on 9-mers, and are thus less accurate). CD4 epitope predictions were done with the NetMHCIIpan 2.1 Server, which predicts binding of peptides to each subject’s MHC class II HLA-DR alleles using artificial neural networks [9] (http://www.cbs.dtu.dk/services/NetMHCIIpan/). Predicted epitopes were 15-mers, and unique epitopes were retained based on the core 8-mer peptide sequence: between several overlapping peptides with an identical core peptide, the peptide that had the strongest predicted binding affinity was retained.

Predictions are given with IC50 values (in nM), with a threshold of 50 nM for an epitope to be considered a strong binder (SB); weak binders (WB) have a predicted IC50 between 50 and 500 nM.

Epitopes were predicted on the translated AA sequences corresponding to Gag, Pol, Env and Nef based on each subject’s class I and II HLA alleles. In parallel, epitopes were predicted in the vaccine insert sequences using all the HLA/HLA-DR alleles in the cohort of 110 RV144 subjects. For each subject, all unique HLA-peptide binding pairs were retained, leading to some peptides being counted multiple times for each HLA to which it was predicted to bind.

Subject-specific epitopes were matched to the vaccine-derived epitopes – epitopes were matched when the subject- and vaccine-derived peptides (with the same HXB2 positions) had at least 67% AA identity, or a maximum of 3 mismatches for a 9-mer; we consider that 9-mers with 4 or more mismatches cannot be aligned with confidence and our rationale for not aligning such 9-mers is that the sequences are too distant for the peptide to be recognized by a vaccine-elicited response [10]. Matched epitope predictions were analyzed based on the respective binding affinity values of the matched epitopes, and on the evolutionary distance calculated between the matched epitopes. For each matched subject-derived and vaccine-derived epitope pair, the binding affinity value was calculated as the ratio of the binding affinity for the subject-derived epitope to the binding affinity of the vaccine-derived epitope. The evolutionary distance was calculated between the subject-derived and vaccine-derived epitope based on the HIV-specific matrix (HIV-between-10%) developed by Nickle and colleagues [11].

For each subject, summary distances were computed based on matched pairs of predicted epitopes (if there were no predicted epitope or no matched pair, then the distance could not be defined and the subject's information was not used). Wilcoxon rank sum tests (equivalently, the Mann-Whitney test) with exact 2-sided p-values were used to test for a different distribution in summary measures between the vaccine and placebo groups.

### Phylogenetic dependency networks

We used phylogenetic dependency networks, a statistical model of evolution that simultaneously takes into account HIV-1 AA co-variation, linkage disequilibrium among HLA alleles, and shared ancestry in the HIV-1 phylogeny to identify the primary source of selection pressure acting on each HIV codon [12]. For each gene, a maximum likelihood phylogenetic tree was constructed and a model of conditional adaptation was created for the vaccine status and for every HLA gene, amino acid position and state. The null hypothesis is that the observations depend on the phylogenetic tree structure; then, adaptation due to each variable is modeled along the tree by an additive process. Results were adjusted for multiple comparisons, using q values of ≤0.2 with an associated p-value threshold of ≤0.05 (implying a false-positive proportion of 20% among identified associations).

### Association between viral loads and HLA alleles

We analyzed the effect of the HLA (2 digit) genotype on viral loads measured at the time of HIV-1 diagnosis (VL corresponding to the plasma sample sequenced).

L1-regularized linear regression analyses were used to test which features predict VL for each sample. A bootstrap procedure (sample with replacement 1,000 times) was used to estimate a bootstrap support frequency for a given predictor and its effect size on viral loads.

## Results

### Gag epitope repertoires from vaccine recipients were smaller than those of placebo recipients

**Figure 1:**
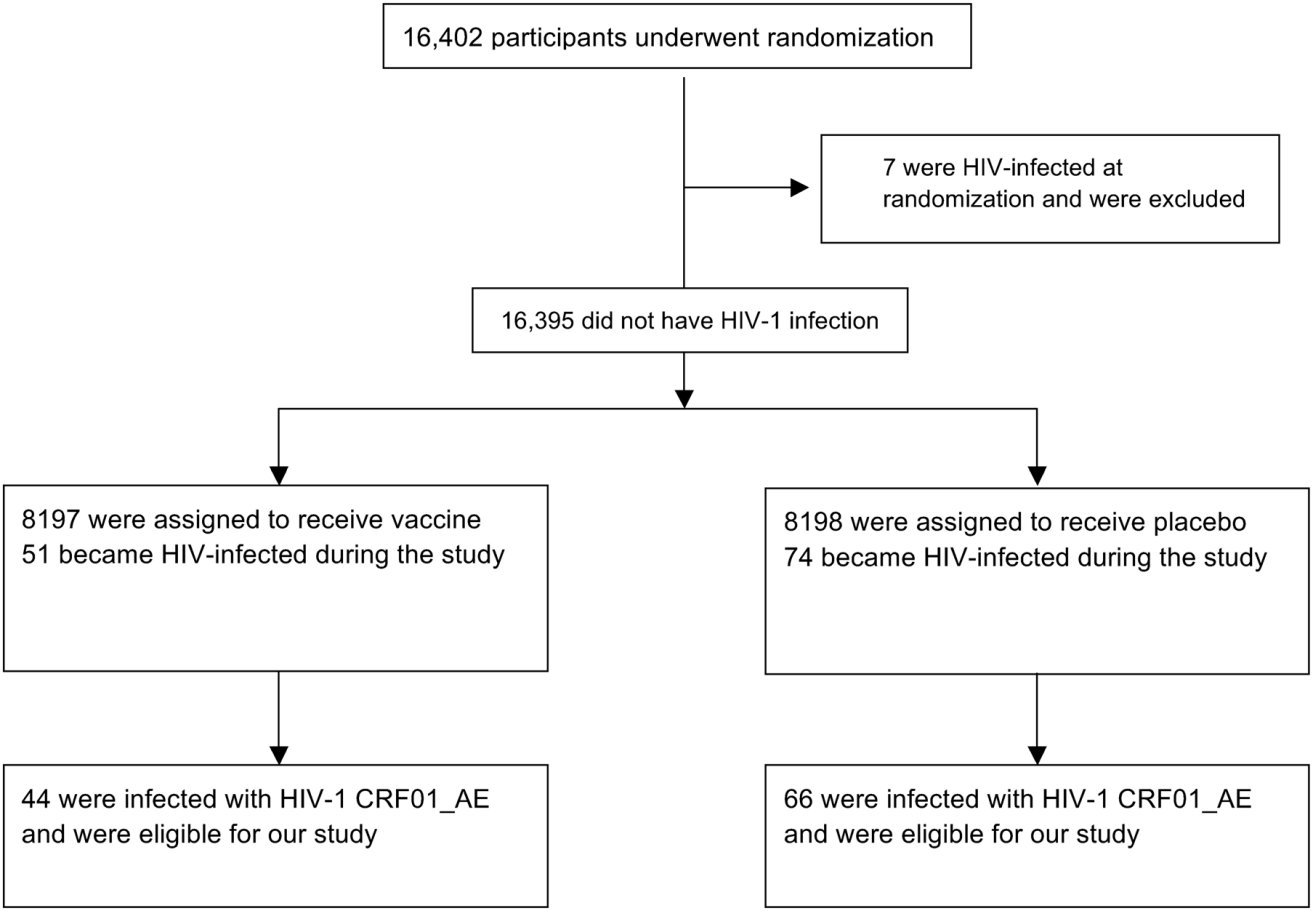
Flowchart diagram of HIV-1 breakthrough infections in RV144.

**Figure 2:**
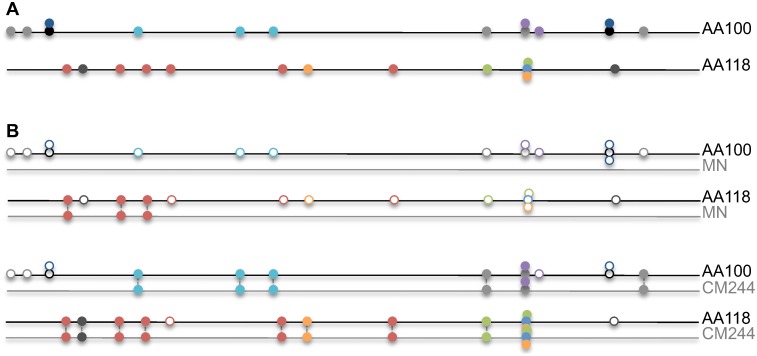
Schematic representation of epitope predictions in RV144 HIV-1 breakthrough sequences, and their comparison to RV144 vaccine inserts. A. Each line represents the Env-gp120 sequence from a subject and each circle a CD8 epitope prediction (different colors for different HLA alleles). The figure represents epitopes predicted based on each subject’s HLA class I genotype for two subjects who were infected with a nearly identical virus (AA100: HLA-A*02∶03, HLA-A*24∶10, HLA-B*18∶01, HLA-B*18∶02, HLA-C*07∶04; AA118: HLA-A*11∶01, HLA-A*24∶07, HLA-B*44∶03, HLA-C*01∶02, HLA-C*07∶01). B. Epitope repertoires from a given subject are compared to the epitope predictions for the vaccine insert sequences (CM244 and MN) based on that subject’s HLA class I genotype. Empty circles represent epitopes predicted in the sequence from a subject that could not be matched to a corresponding epitope prediction based on the vaccine insert sequence and the subject’s HLA class I genotype. More subject-derived epitopes were matched against the vaccine insert CM244 than against MN; both subjects were infected by a CRF01-AE virus like CM244, while MN is a subtype B virus.

We performed CD8 and CD4 epitope predictions based on each subject’s genotype and HIV-1 genomic sequences derived at the time of HIV-1 diagnosis. To avoid the misidentification of effects that would be due to a phylogenetic difference, we only included the 110 subjects infected with CRF01_AE viruses – see Flowchart on Figure 1. For these 44 vaccine and 66 placebo recipients, the vaccine efficacy was estimated at 34% (95% C.I. = 7.8%, 54.7%) (compared to 31% in the full mITT cohort [1]), thus allowing vaccine/placebo investigations of the impact of the vaccine. Epitope predictions from two subjects in a linked HIV-1 transmission were included since, despite being infected with nearly identical viruses, both subjects had different HLA genotypes and thus non-overlapping predicted epitope repertoires (Figure 2A).

The number of predicted epitopes for each subject depends on both the genotype of the individual and the HIV-1 sequence they were infected with. There was no difference in the distribution of HLA types between the vaccine and placebo groups; then we looked at the epitopes predicted for these HLA alleles. When the vaccine and placebo groups were compared, there was no difference in the number of predicted CD8 epitopes in Pol, Env and Nef: Median number of epitopes in Pro: n = 5 (vaccine) and n = 6 (placebo), p = 0.464; in RT-IN: n = 116 (vaccine) and n = 117.5 (placebo), p = 0.707; in Env: n = 64 (vaccine) and n = 66 (placebo), p = 0.238; in Nef: n = 25 (vaccine) and n = 26.5 (placebo), p = 0.408 (Table 1). In contrast, there were significantly fewer Gag epitopes predicted in sequences from vaccine recipients (n = 45) than in those from placebo recipients (n = 51.5), p = 0.019 (Table 1).

**Table 1:** Number of CD8 epitopes predicted for each subject depending on his HLA type.

	Gag		Pro		Env	
	Placebo	Vaccine	Placebo	Vaccine	Placebo	Vaccine
Nr of subjects	66	44	65	43	66	44
Median	51.5	45	6	5	66	64
Mean	52.71	46.11	6.39	6.21	69.68	64.75
p-value	0.019		0.464		0.238	
	**RT-IN**		**Nef**			
	**Placebo**	**Vaccine**	**Placebo**	**Vaccine**		
Nr of subjects	66	44	66	44		
Median	117.5	116	26.5	25		
Mean	116.30	113.80	26.42	25.30		
p-value	0.707		0.408			

Predictions are given for proteins that corresponded to the vaccine inserts (Gag, Pro, Env), and for proteins that were not part of the vaccine (RT-IN, and Nef).

### Env epitopes from RV144 participants poorly matched vaccine-derived epitopes

We can hypothesize that, due to vaccine-engendered escape mutations, fewer epitopes may have a corresponding matched epitope in the vaccine insert sequence in sequences from vaccine recipients than in those from placebo recipients. For each subject, the list of predicted autologous class I and class II epitopes was compared to corresponding epitopes predicted in the vaccine insert strain to identify pairs of matched subject+vaccine epitopes (Schematic representation for class I epitopes in Figure 2B). Considering each vaccine insert sequence separately, we found no difference between vaccine and placebo recipients in the ratio of subject-derived epitopes that could be matched to vaccine-derived epitopes for any of the HIV-1 proteins: p≥0.314 vs CM244, p≥0.214 vs LAI (MN for Env), p≥0.540 vs 92TH023. Since the RV144 vaccine was composed of proteins of different subtype (Subtype B and CRF01_AE; while all subjects evaluated were infected with CRF01_AE) and of proteins that are relatively variable (Env-gp120) or conserved (Gag/Pro), we tested if these factors affected the ability to match predicted autologous epitopes to the predicted epitopes in the inserts. We found that the ratio of matched epitopes differed depending on the protein and on the vaccine reference considered but not on the vaccine/placebo status. The proportion of matched epitopes is much higher in Pol, Gag, or Nef (73 to 91%) than in Env, for which only about a third (36%) of predicted epitopes in subject-derived sequences could be matched to epitopes identified in the vaccine inserts (Table 2). In addition, there were subtype-specific differences, with significantly more subject-derived epitopes matched against the CM244 (also CRF01) than against the MN (or LAI; both subtype B) vaccine insert. For example, an average of 55% of Env predicted epitopes were matched against CM244 compared to 7% matched against MN (p<0.0001) whether vaccine or placebo recipients are considered. The limited number of matched epitopes is due to the high diversity between Env sequences, hence the large distance between specific strains, which is amplified when attempting cross-subtype epitope matching.

**Table 2:** Subject-specific epitopes matched to vaccine-derived epitopes.

Matched epitopes	Gag	Pol	Env	Nef
CD8	4,629/5,509	12,198/13,368	5,545/15,520	2,389/2,860
CD8 (%)	84%	91%	36%	84%
CD4	11,177/13,419	25,273/29,921	13,109/36,738	3,006/4,119
CD4 (%)	83%	84%	36%	73%

Epitopes were considered matched when the subject- and vaccine-derived peptides had at least 67% AA identity. Number and percentages of matched epitopes are given for all of the 110 subjects in the cohort; there was no difference between the vaccine and placebo groups.

### No vaccine/placebo distinction in Gag, Pro, Gp120 epitope comparisons against the vaccine inserts

Under the hypothesis that vaccine-induced immune responses could lead to escape mutations in sequences from vaccinees, we used epitopes predicted based on each subject’s genotype to test whether epitope changes relative to the vaccine inserts differed between the vaccine and placebo groups. Because the vaccine was multivalent, we compared the epitopes predicted in RV144 participants’ sequences to the subtype B and CRF01_AE reference strains included in the vaccine (CRF01_AE: CM244 and 92TH023; subtype B: LAI and MN). We tested both differences in binding affinities (by comparing predicted IC50 for the epitopes in the vaccinee’s sequence and in the vaccine insert) and in evolutionary distances (i.e., protein distances between RV144 participants’ epitopes and vaccine-derived epitopes were calculated using an HIV-specific substitution model). We looked for evidence of a difference between vaccine and placebo group by analyzing predictions in proteins that were part of the vaccine (Gag, Pro, Gp120) and used as control the epitope predictions derived from RT-IN and Nef (to test the hypothesis that there would be no difference between the vaccine and placebo group outside of the vaccine insert).

We first looked at predicted CD8 and CD4 epitopes defined as both weak and strong binders. We found no difference between the vaccine and placebo group either when we focused on binding affinities or evolutionary distances for the different proteins and references considered (data not shown). Second, we focused on the subset of epitopes identified as strong binders (i.e., with a predicted IC50≤50 nM). Again, there was no concordant evidence of a difference between the vaccine and placebo group, although, for CD8 epitope predictions, there was a trend suggesting a difference between the vaccine and placebo groups in Pro (p = 0.080) and gp120 (p = 0.065) when binding affinities of predicted epitopes were considered (Table 3). In addition, when the analysis was limited to V2, some results were significant (0.022≤p-value≤0.231; see below). Comparisons of epitope predictions in RT-IN and Nef verified that there was also no distinction between the vaccine and placebo group for proteins not included in the vaccine (p-values≥0.16 for both CD4 and CD8 predictions).

**Table 3:** CD8 and CD4 epitopes predicted to be strong binders matched against different vaccine inserts.

CD8 epitope predictions - Binding affinity						
**Gag**	**P CM244 (66)**	**V CM244 (44)**	**P LAI (66)**	**V LAI (44)**		
Mean	0.996	0.965	0.979	0.979		
p-value	0.266		0.813			
**Pro**	**P CM244 (31)**	**V CM244 (20)**	**P LAI (31)**	**V LAI (20)**		
Mean	0.24	0.338	0.426	0.354		
p-value	0.08		0.333			
**gp120**	**P CM244 (66)**	**V CM244 (44)**	**P MN (66)**	**V MN (44)**	**P 92TH (66)**	**V 92TH (44)**
Mean	0.95	0.997	0.623	0.676	0.826	0.864
p-value	0.065		0.468		0.448	
**V2**	**P CM244 (58)**	**V CM244 (35)**	**P MN (0)**	**V MN (0)**	**P 92TH (58)**	**V 92TH (35)**
Mean	0.957	1.123	n.a.	n.a.	0.017	0.114
p-value	0.022		n.a.		0.047	
**V3**	**P CM244 (13)**	**V CM244 (9)**	**P MN (0)**	**V MN (0)**	**P 92TH (0)**	**V 92TH (0)**
Mean	0.903	0.548	n.a.	n.a.	n.a.	n.a.
p-value	0.108		n.a.		n.a.	
**RT-In**	**P CM244 (66)**	**V CM244 (44)**	**P LAI (66)**	**V LAI (44)**		
Mean	1	1	0.439	0.641		
p-value	n.a.		0.378			
**Nef**	**P CM244 (66)**	**V CM244 (44)**	**P LAI (66)**	**V LAI (44)**		
Mean	0.98	0.9	0.669	0.635		
p-value	0.162		0.958			
**CD8 epitope predictions - Evolutionary distance**						
**Gag**	**P CM244 (66)**	**V CM244 (44)**	**P LAI (66)**	**V LAI (44)**		
Mean	0.033	0.051	0.142	0.139		
p-value	0.351		0.998			
**Pro**	**P CM244 (31)**	**V CM244 (20)**	**P LAI (31)**	**V LAI (20)**		
Mean	0.124	0.14	0.152	0.113		
p-value	0.851		0.361			
**gp120**	**P CM244 (66)**	**V CM244 (44)**	**P MN (66)**	**V MN (44)**	**P 92TH (66)**	**V 92TH (44)**
Mean	0.056	0.064	0.005	0.005	0.014	0.016
p-value	0.403		0.356		0.859	
**V2**	**P CM244 (58)**	**V CM244 (35)**	**P MN (0)**	**V MN (0)**	**P 92TH (58)**	**V 92TH (35)**
Mean	0.095	0.101	n.a.	n.a.	0.049	0.025
p-value	0.058		n.a.		0.231	
**V3**	**P CM244 (13)**	**V CM244 (9)**	**P MN (0)**	**V MN (0)**	**P 92TH (0)**	**V 92TH (0)**
Mean	0.21	0.148	n.a.	n.a.	n.a.	n.a.
p-value	0.227		n.a.		n.a.	
**RT-In**	**P CM244 (66)**	**V CM244 (44)**	**P LAI (66)**	**V LAI (44)**		
Mean	0.002	0.002	0.038	0.03		
p-value	0.792		0.616			
**Nef**	**P CM244 (64)**	**V CM244 (44)**	**P LAI (64)**	**V LAI (44)**		
Mean	0.081	0.087	0.281	0.247		
p-value	0.803		0.458			
**CD4 epitope predictions - Binding affinity**						
**Gag**	**P CM244 (62)**	**V CM244 (43)**	**P LAI (62)**	**V LAI (43)**		
Mean	0.991	0.985	1.002	0.948		
p-value	0.688		0.296			
**Pro**	**P CM244 (40)**	**V CM244 (22)**	**P LAI (40)**	**V LAI (22)**		
Mean	0.816	0.804	0.897	0.971		
p-value	0.794		0.662			
**gp120**	**P CM244 (59)**	**V CM244 (43)**	**P MN (59)**	**V MN (43)**	**P 92TH (59)**	**V 92TH (43)**
Mean	0.86	0.847	0.548	0.374	0.528	0.4
p-value	0.989		0.127		0.336	
**V2**	**P CM244 (45)**	**V CM244 (22)**	**P MN (45)**	**V MN (22)**	**P 92TH (45)**	**V 92TH (22)**
Mean	1.001	1.14	n.a.	n.a.	0	0.045
p-value	0.177		n.a.		0.162	
**V3**	**P CM244 (15)**	**V CM244 (4)**	**P MN (15)**	**V MN (4)**	**P 92TH (15)**	**V 92TH (4)**
Mean	0.314	0.259	n.a.	n.a.	n.a.	n.a.
p-value	0.881		n.a.		n.a.	
**RT-In**	**P CM244 (62)**	**V CM244 (43)**	**P LAI (62)**	**V LAI (43)**		
Mean	0.999	1	0.689	0.6		
p-value	0.416		0.403			
**Nef**	**P CM244 (54)**	**V CM244 (35)**	**P LAI (54)**	**V LAI (35)**		
Mean	1.001	1.044	0.28	0.201		
p-value	0.202		0.186			
**CD4 epitope predictions - Evolutionary distance**						
**Gag**	**P CM244 (61)**	**V CM244 (43)**	**P LAI (61)**	**V LAI (43)**		
Mean	0.021	0.025	0.112	0.089		
p-value	0.806		0.04			
**Pro**	**P CM244 (40)**	**V CM244 (22)**	**P LAI (40)**	**V LAI (22)**		
Mean	0.111	0.076	0.121	0.095		
p-value	0.299		0.732			
**gp120**	**P CM244 (59)**	**V CM244 (43)**	**P MN (59)**	**V MN (43)**	**P 92TH (59)**	**V 92TH (43)**
Mean	0.097	0.083	0.025	0.015	0.046	0.035
p-value	0.108		0.12		0.195	
**V2**	**P CM244 (45)**	**V CM244 (22)**	**P MN (0)**	**V MN (0)**	**P 92TH (45)**	**V 92TH (22)**
Mean	0.168	0.089	n.a.	n.a.	0.041	0.047
p-value	0.01		n.a.		0.602	
**V3**	**P CM244 (15)**	**V CM244 (4)**	**P MN (0)**	**V MN (0)**	**P 92TH (15)**	**V 92TH (4)**
Mean	0.235	0.2	n.a.	n.a.	0.043	0.176
p-value	0.96		n.a.		0.272	
**RT-In**	**P CM244 (62)**	**V CM244 (43)**	**P LAI (62)**	**V LAI (43)**		
Mean	0.006	0.002	0.035	0.035		
p-value	0.332		0.65			
**Nef**	**P CM244 (54)**	**V CM244 (35)**	**P LAI (54)**	**V LAI (35)**		
Mean	0.107	0.105	0.347	0.405		
p-value	0.956		0.296			

Epitopes were predicted in all HIV-1 proteome sequences derived from RV144 breakthrough infections. The epitopes were matched against epitopes derived from the RV144 vaccine inserts of subtype B (MN, LAI) or CRF01_AE (CM244, 92TH023); two epitope characteristics were used to compare epitopes from the breakthrough to the vaccine: the predicted binding affinity for each epitope and the protein distance between the epitope sequences. One summary measure was computed for each protein and each subject, and comparisons were done between the vaccine (V) and placebo (P) groups (the number of vaccine and placebo recipients included in each group is in parenthesis) with Mann-Whitney tests for proteins corresponding to those included in the RV144 vaccine insert (Gag, Pro, gp120 (including V2)) and those not part of the RV144 vaccine (RT-IN, Nef).

### Env-V2 and Env-V3-specific comparisons of epitope predictions

Given the results of the RV144 correlates of risk study [4], a V2-specifc analysis was performed. While analyses of CD8 epitope predictions that included both strong and weak binders showed that only the comparison of V2-binding affinity measures against CM244 was significant (p = 0.017), the results were more consistent when we focused on the subset of epitopes identified as strong binders (i.e., with a predicted IC50≤50 nM) (Table 3). Data were not available against the subtype B vaccine boost (MN) because subject-derived epitopes were too divergent to be matched to the vaccine strain. For predicted strong CD8 binders, there was a significant difference between the vaccine and placebo group in the V2 region of Env when binding affinities were considered. Comparisons were significant against the CRF01_AE strains (CM244 p = 0.022; 92TH023 p = 0.047). When evolutionary distances were considered for CD8 epitopes, there was only a trend suggesting a difference between the vaccine and placebo group against the CRF01_AE strains (CM244 p = 0.058; 92TH023 p = 0.231).

When CD4 epitope predictions were considered, there were weak trends suggesting a greater number of strong binders in the V2 of vaccinees compared to the placebo group (CM244 p = 0.177; 92TH023 p = 0.162), and significantly greater evolutionary distances in placebo recipients against CM244 (p = 0.010), but not against 92TH023 (p = 0.602).

Given that high responses to V3 have been associated with a lower risk of HIV-1 infection among vaccinees who had low gp120-specific plasma IgA: (OR = 0.49, p = 0.007), we also performed a V3-specific analysis for CD8 and CD4 epitopes. There was no significant distinction found between the vaccine and placebo groups using the tests described above (p>0.108), noting that there were no epitopes in V3 that had enough similarity with MN for the corresponding epitopes to be matched (i.e., less than 2/3 of the residues of a peptide matched).

**Figure 3:**
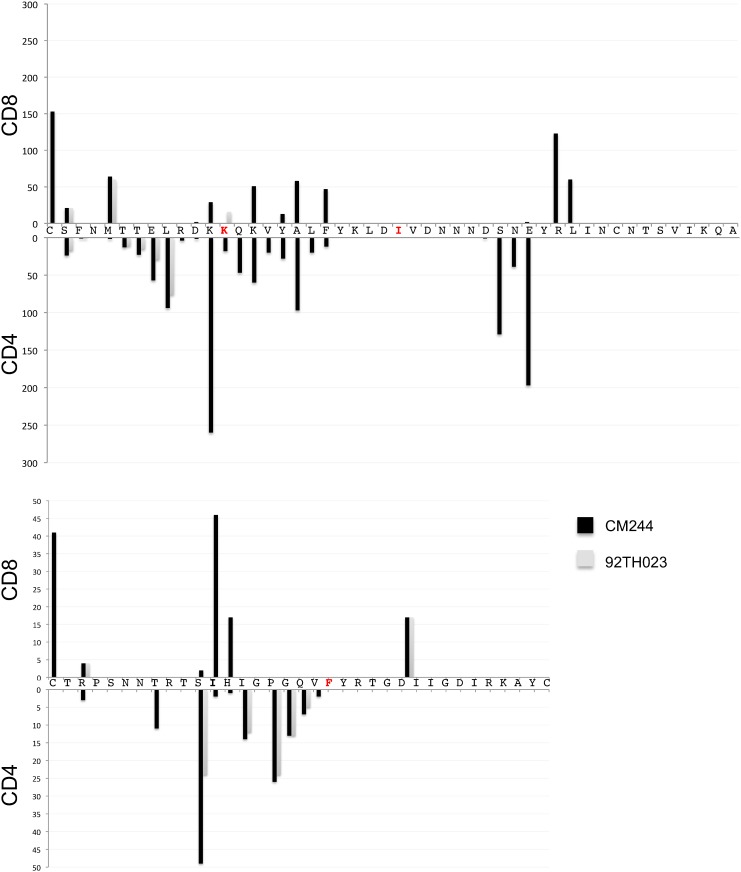
Overlap between predicted CD8 and CD4 epitopes in Env-V2 and -V3 in sequences from HIV-1-infected RV144 participants. The x-axis corresponds to the V2 and V3 sequence, and the y-axis corresponds to the number of predicted epitopes starting at each position. The epitope predictions correspond to all unique HLA/peptide combinations, hence the number of epitope predictions starting at a specific location can surpass the number of subjects in the cohort because a given peptide can be predicted as an epitope for multiple HLA alleles. The amino acids in red correspond to sites that were identified as genetic signatures that distinguished breakthrough sequences from vaccine and placebo recipients.

Parallels between V2 and V3 were notable: i) both V2 and V3 were a hotspot of RV144-induced antibody responses [13]; ii) V2- and V3-specific binding antibodies were identified as correlates of risk of infection in RV144; and iii) they harbored signature sites of RV144 vaccination. Statistical analyses that interrogated individual sites in Env from RV144 participants identified two sites in V2 (sites 169 and 181, [2]) and one in V3 (site 317). Thus, we looked specifically at predicted epitopes in V2 and V3 that spanned the signature sites. Figure 3 shows the overlap between CD8 and CD4 predicted epitopes in V2 and V3: we note that because multiple alleles can restrict the same peptide the number of epitope predictions starting at a specific location can surpass the number of subjects in the cohort. In addition, there are epitopes spanning the signature sites in both V2 and V3; however, no distinction was detected between the vaccine and placebo group (Table S1 in File S2).

### No allele-specific effect in vaccine/placebo epitope comparisons against the vaccine inserts

By analyzing Env sequence data as a function of each subject’s genotype on a site-by-site basis with a phylogenetically-corrected method, five DRB1 and one class I HLA alleles (DRB1*03, DRB1*04, DRB1*07, DRB1*11, DRB1*15, A*68) were associated with specific AA polymorphisms in Env. However, the limited number of subjects with a given allele (ranging from four with DRB1*11 to 33 with DRB1*15) did not allow us to perform meaningful vaccine/placebo comparisons restricted to carriers of these specific alleles.

Next, when log_10_ viral loads (measured at the time of sampling for viral genome sequencing, corresponding to early infection as the last negative visit happened six months prior diagnosis) were analyzed as a function of Env sequence together with each subject’s genotype, HLA alleles HLA-A*11 and HLA-B*46 were associated with higher viral loads, although the effect sizes were small (HLA-A*11: weight = 0.23, bootstrap support = 0.87; HLA-B*46: weight = 0.12, bootstrap support = 0.70). We therefore examined epitope metrics for carriers of HLA-A*11 (n = 59), HLA-B*46 (n = 40), as well as for carriers of HLA-A*02 as this is another frequent allele in the RV144 cohort (n = 53). We repeated the analysis described above by taking into account separately the predicted epitopes restricted by HLA-A*02, HLA-A*11, and HLA-B*46; including strong and weak binders (focusing only on strong binders reduced the subset of predicted epitopes to a number too small for adequate comparisons).

For these allele-specific comparisons, there was no difference between the vaccine and placebo groups whether evolutionary distances or binding affinity were considered – the smallest p-values were p = 0.206 for evolutionary distances (gp120 vs CM244 for HLA-A*02), and p = 0.123 for binding affinity measures (V2 vs CM244 for HLA-B*46) (Table S2 in file S2). There was a p-value = 0.045 for Nef epitope predictions restricted by HLA-A*11; this result should probably not be viewed as a significant vaccine/placebo distinction given the high number of tests performed, that Nef was not part of the vaccine, and that it is a comparison against the cross-subtype reference (subtype B) while all subjects analyzed here were infected with CRF01_AE viruses.

### No relationship between epitopic distances and the duration of HIV-1 infection

We looked at the relationship between the evolutionary distance (defined above) and the mean diversity in each subject, the latter of which can be used as a measure of the age of the infection. The hypothesis is that if the epitope distances track intra-host diversity it could be interpreted as a sign of intra-host evolution, i.e., a post-infection effect. We found no relationship between the epitopic evolutionary distance and the mean diversity in each subject: For V2 CD8 predictions: Spearman correlation coefficient ρ = –0.113 (p = 0.283); for V2 CD4 predictions: Spearman correlation coefficient ρ = 0.011 (p = 0.929). There was also no relationship between the epitopic evolutionary distance and the number of days since the last negative visit in each subject (V2 CD8 predictions: Spearman correlation coefficient ρ = –0.188 (p = 0.071); V2 CD4 predictions: Spearman correlation coefficient ρ = –0.075 (p = 0.544)).

## Discussion

Here we analyzed epitope predictions derived from HIV-1 genome sequences corresponding to 110 CRF01_AE breakthrough infections in the RV144 trial, including 44 vaccine and 66 placebo recipients. We tested for evidence of a distinction between the vaccine and placebo groups and found evidence potentially suggestive of a weak T cell driven sieve effect among breakthrough viruses as vaccine/placebo comparisons showed a) some evidence of a signal converging on the Env-V2 segment, and b) smaller Gag epitope repertoires in vaccine recipients compared to placebo recipients.

One indication that the V2 signal, although weak, may be genuine is the fact that differences between the vaccine and placebo group were only seen when comparisons were made against CRF01_AE strains (CM244, 92TH023). No difference was seen when subject-derived sequences were compared to epitopes derived from the subtype B MN strain; this appears logical as the MN boost protein would seem unlikely to have elicited a substantial number of cross-reactive T cell responses toward infecting CRF01_AE viruses, which at the epitope level often differed from MN by 4–5 residues out of nine in a CTL epitope. The lack of V2 signal against MN as a reference could be expected as cross-reactivity decreases drastically with more than 2 mutations out of 9 residues in a CTL epitope [10] - a typical instance when epitopes are compared between subtype B and CRF01_AE. An additional factor that may explain the identification of a vaccine/placebo distinction in V2 is the fact that 60% of the CD4 responses detected in a subset of RV144 vaccine recipients were directed against V2 [14], although, paradoxically, these responses were not identified post-infection, suggesting that antigen-specific T cells could possibly have been preferentially infected and deleted [15]. Importantly, it is possible that the signal detected in V2 was the consequence of an antibody-mediated effect, as binding antibodies targeting V2 were associated with a decreased risk of HIV-1 infection in the RV144 trial. As such, two vaccine-associated signatures in V2 [2] that were linked to vaccine-derived binding antibodies [3] were located within predicted CD8/4 epitopes. Overall, it is difficult to hypothesize that T cell driven immune responses played an important role in the protection associated with the RV144 vaccine because few RV144 subjects mounted CD8/CD4 responses following RV144 vaccination [1]. However, we cannot discount that T cell responses may have played a role in a more restricted way as the limited sensitivity of the T cell assays employed in the RV144 trial means that some responses may not have been detected.

Our results were generally, but not necessarily, in agreement between the CD8 and CD4 epitope predictions. It has to be noted that CD4 epitopes are less well defined than CD8 epitopes in the context of HIV-1 natural infection, hence *in silico* predictions of CD4 epitopes are less accurate than those for CD8 epitopes. It is possible that the absence of information and algorithms for the prediction of epitopes in HLA class II alleles common in Thailand may underestimate epitope enumeration and bias this analysis. To overcome the uncertainty in epitope predictions, additional analyses were focused on a few V2 epitopes that had been precisely characterized in some RV144 participants ([16]; and personal communication from Mark deSouza and Silvia Ratto-Kim). For example, we specifically looked at predicted epitopes corresponding to the peptide that showed the highest recognition in a study of responses from 25 RV144 vaccine recipients following vaccination: peptide VHALFYKLDIVPIED (AA 172–186) [16]. However, these epitope comparisons focused on the experimental data showed no distinction between vaccine and placebo groups, while this may be due in part to the small number of subjects, it also illustrates the limited congruence between *in silico* predictions and mapped CTL responses, as previously noted [6].

The second signal we identified was the significantly (p = 0.019) smaller size of Gag epitope repertoires among vaccine recipients compared to placebo recipients. One interpretation is that there could be more escape mutations in Gag sequences from vaccinees (due to vaccine-induced responses), and that these mutations prevented certain sequence motifs from being recognized as epitopes. Another interpretation is that vaccine efficacy might depend on the number of Gag epitopes in the circulating viruses, implying that viruses with canonical epitopes were more likely to be blocked from establishing infection. Some evidence of a vaccine/placebo distinction in Gag is interesting as Gag is typically a preferential target of T cell responses compared to the V2 region of Env. Indeed, a T cell driven sieve effect may have been expected to be uncovered in an immunodominant region of the proteome, as was shown in a study of the Step trial [6]. Analyses of epitope repertoires from breakthrough infections in the Step trial showed a distinction between the vaccine and placebo group that was largely driven by an effect in Gag. Several factors allowed for the detection of a T cell based signal in Gag in the Step trial: i) most subjects mounted T cell responses following vaccination; ii) there are immunodominant responses in Gag; iii) a Gag immunodominant response (SL9) was restricted by a common allele in the Step cohort (HLA-A*02); iv) Gag is a relatively conserved protein allowing for an easier identification of genetic signals than a more variable protein (such as Env); v) there was no env immunogen to potentially shift immunodominance patterns in vaccinees.

Lastly, our results differ from those of Gartland and colleagues [17], who reported evidence of greater predicted HLA binding escape for an HLA A*02 peptide in vaccine versus placebo recipients, and greater vaccine efficacy in A*02-positive participants than in A*02-negative participants (VE = 54% versus 3%, P = 0.05). We note that a comprehensive test of all HLA alleles in the RV144 cohort failed to show that A*02 modified vaccine efficacy. The differences between our studies could also be due to the fact that the MN-derived V2 peptide linked to the findings by Gartland and colleagues was not included as a predicted epitope in our analysis, because we only considered as a predicted epitope the peptides that had a predicted binding affinity <500 nm (different methods can be used to analyze sequences without conditioning on the presence of potential epitopes [18]). Besides, few MN-derived epitopes were included in our cross-proteome analysis since subtype B- (MN vaccine insert) and CRF01_AE- (breakthrough viruses) derived epitopes were poorly matched; the MN peptide highlighted in [17] showed three to six AA differences with corresponding breakthrough-derived peptides, suggesting a limited probability of cross-reactive CTL responses with the breakthrough peptides (knowing that the MN component of the RV144 vaccine was a protein boost, which would not typically be expected to elicit CTL responses).

In conclusion, while there is some evidence that T cell driven immune responses may have been associated with genetic changes in HIV-1 breakthrough viruses from vaccinees, the fact that most results are not strongly corroborated across our proteome-wide epitope comparisons is consistent with weak CD8-driven cytotoxic T cell responses in RV144. Hence, in the absence of evidence for strong CD8+ T cell epitopic signals across the RV144 vaccine inserts, the most conservative interpretation of our findings for V2 epitope predictions would suggest a relationship to V2-specific binding antibody responses previously identified as a correlate of risk in RV144 [4].

## Supporting Information

File S1 **File S1 corresponds to the RV144 protocol (v3_7, from July 27, 2008).**
(PDF)

File S2 **File S2 includes tables S1 and S2.** Table S1 corresponds to epitope predictions limited to the Env-V2/V3 region and Table S2 to predictions for specific HLA alleles.
(XLSX)

## References

[1] Rerks-Ngarm S, Pitisuttithum P, Nitayaphan S, Kaewkungwal J, Chiu J, et al (2009) Vaccination with ALVAC and AIDSVAX to prevent HIV-1 infection in Thailand. N Engl J Med 361: 2209–2220. 19843557 10.1056/NEJMoa0908492

[2] Rolland M, Edlefsen PT, Larsen BB, Tovanabutra S, Sanders-Buell E, et al (2012) Increased HIV-1 vaccine efficacy against viruses with genetic signatures in Env V2. Nature. 10.1038/nature11519 PMC3551291 22960785

[3] Liao HX, Bonsignori M, Alam SM, McLellan JS, Tomaras GD, et al (2013) Vaccine induction of antibodies against a structurally heterogeneous site of immune pressure within HIV-1 envelope protein variable regions 1 and 2. Immunity 38: 176–186. 23313589 10.1016/j.immuni.2012.11.011 PMC3569735

[4] Haynes BF, Gilbert PB, McElrath MJ, Zolla-Pazner S, Tomaras GD, et al (2012) Immune-correlates analysis of an HIV-1 vaccine efficacy trial. N Engl J Med 366: 1275–1286. 22475592 10.1056/NEJMoa1113425 PMC3371689

[5] Rolland M, Heckerman D, Deng W, Rousseau C, Coovadia H, et al (2008) Broad and Gag-biased HIV-1 epitope repertoires are associated with lower viral loads. PLoS ONE 3: e1424. 18183304 10.1371/journal.pone.0001424 PMC2170517

[6] Rolland M, Tovanabutra S, deCamp AC, Frahm N, Gilbert PB, et al (2011) Genetic impact of vaccination on breakthrough HIV-1 sequences from the STEP trial. Nat Med 17: 366–371. 21358627 10.1038/nm.2316 PMC3053571

[7] Hurley CK, Mack SJ, Mickelson E, Marsh S, Tilanus MGJ, et al. (2006) HLA typing and informatics. in immunobiology of the human MHC, in J A Hansen (ed), 13th International Histocompatibility Workshop protocols IHWG Press, Seattle, WA: 179–352.

[8] Nielsen M, Lundegaard C, Blicher T, Lamberth K, Harndahl M, et al (2007) NetMHCpan, a method for quantitative predictions of peptide binding to any HLA-A and -B locus protein of known sequence. PLoS ONE 2: e796. 17726526 10.1371/journal.pone.0000796 PMC1949492

[9] Nielsen M, Justesen S, Lund O, Lundegaard C, Buus S (2010) NetMHCIIpan-2.0 - Improved pan-specific HLA-DR predictions using a novel concurrent alignment and weight optimization training procedure. Immunome Res 6: 9. 21073747 10.1186/1745-7580-6-9 PMC2994798

[10] Rolland M, Frahm N, Nickle DC, Jojic N, Deng W, et al (2011) Increased breadth and depth of cytotoxic T lymphocytes responses against HIV-1-B Nef by inclusion of epitope variant sequences. PLoS ONE 6: e17969. 21464919 10.1371/journal.pone.0017969 PMC3065451

[11] Nickle DC, Heath L, Jensen MA, Gilbert PB, Mullins JI, et al (2007) HIV-specific probabilistic models of protein evolution. PLoS One 2: e503. 17551583 10.1371/journal.pone.0000503 PMC1876811

[12] Carlson JM, Brumme ZL, Rousseau CM, Brumme CJ, Matthews P, et al (2008) Phylogenetic dependency networks: inferring patterns of CTL escape and codon covariation in HIV-1 Gag. PLoS Comput Biol 4: e1000225. 19023406 10.1371/journal.pcbi.1000225 PMC2579584

[13] Karasavvas N, Billings E, Rao M, Williams C, Zolla-Pazner S, et al (2012) The Thai Phase III HIV Type 1 Vaccine trial (RV144) regimen induces antibodies that target conserved regions within the V2 loop of gp120. AIDS Res Hum Retroviruses 28: 1444–1457. 23035746 10.1089/aid.2012.0103 PMC3484815

[14] de Souza MS, Ratto-Kim S, Chuenarom W, Schuetz A, Chantakulkij S, et al (2012) The Thai phase III trial (RV144) vaccine regimen induces T cell responses that preferentially target epitopes within the V2 region of HIV-1 envelope. J Immunol 188: 5166–5176. 22529301 10.4049/jimmunol.1102756 PMC3383859

[15] Douek DC, Betts MR, Hill BJ, Little SJ, Lempicki R, et al (2001) Evidence for increased T cell turnover and decreased thymic output in HIV infection. J Immunol 167: 6663–6668. 11714838 10.4049/jimmunol.167.11.6663

[16] de Souza MS, Ratto-Kim S, Chuenarom W, Schuetz A, Chantakulkij S, et al (2012) The Thai phase III trial (RV144) vaccine regimen induces T cell responses that preferentially target epitopes within the V2 region of HIV-1 envelope. Journal of immunology 188: 5166–5176. 10.4049/jimmunol.1102756 PMC3383859 22529301

[17] Gartland AJ, Li S, McNevin J, Tomaras GD, Gottardo R, et al (2014) Analysis of HLA A*02 Association with Vaccine Efficacy in the RV144 HIV-1 Vaccine Trial. Journal of virology 88: 8242–8255. 24829343 10.1128/JVI.01164-14 PMC4135964

[18] Edlefsen PT, Gilbert PB, Rolland M (2013) Sieve analysis in HIV-1 vaccine efficacy trials. Curr Opin HIV AIDS 8: 432–436. 23719202 10.1097/COH.0b013e328362db2b PMC3863593

